# Sleep Deprivation and Fatigue in Healthcare Staff: A Clinical Audit on the Risk to Patient Safety

**DOI:** 10.7759/cureus.96543

**Published:** 2025-11-11

**Authors:** Adanna Chukwunonso-Ogbu, Shah Ahmad Fazli, Godino Kalungi, Oladeji Malomo

**Affiliations:** 1 Orthopedic Surgery, Frimley Health NHS Foundation Trust, Frimley, GBR; 2 Internal Medicine, Frimley Health NHS Foundation Trust, Frimley, GBR; 3 General Surgery, Frimley Health NHS Foundation Trust, Frimley, GBR

**Keywords:** fatigue in healthcare staff, healthcare staff, lack of sleep, protected break and break rooms, quality improvement and patient safety

## Abstract

Background and objective

Restful sleep is essential, as it helps recharge the entire being. The risk that sleep deprivation and fatigue among healthcare staff pose to patient safety is often overlooked, which can be detrimental to patient safety and outcomes. Prolonged shifts, night duties, and inadequate rest all contribute to fatigue, impair clinical judgment, and increase the likelihood of errors. This research aims to assess the prevalence of sleep deprivation and fatigue among healthcare professionals, examine its association with patient safety incidents, and provide recommendations to mitigate fatigue-related risks in high-acuity clinical settings.

Methods

This cross-sectional, mixed-method study was conducted over three months across three high-turnover hospitals under the same trust. Quantitative data were collected through an anonymous staff survey assessing average sleep duration, frequency of missed breaks, and fatigue-related cognitive symptoms. Qualitative responses were analyzed to identify recurring themes related to fatigue culture and systemic barriers. The study was conducted in high-acuity hospitals within the same UK NHS trust, in three different locations characterized by high patient turnover and complex medical needs. Staff in these settings routinely work long shifts, including overnight duties and back-to-back rotas. Participants included clinical staff working in the selected wards during the three-month data collection period. This included registered nurses, junior doctors, healthcare assistants, and allied health professionals. A total of 102 staff completed the anonymous questionnaire, and 87 participated in structured face-to-face interviews. The primary outcome measure was the proportion of staff reporting less than six hours of sleep before their clinical shift. Secondary outcomes included the frequency of missed or interrupted breaks and self-reported instances of fatigue-related performance issues.

Results

Thirty-eight percent of staff reported sleeping less than six hours before a shift. Forty-four percent indicated they frequently missed protected breaks, and 100% acknowledged experiencing fatigue-related performance lapses. Thirty-nine percent of staff confirmed witnessing or being involved in a fatigue-related clinical error, while 16% chose not to disclose. Qualitative feedback revealed normalization of exhaustion, reluctance to report fatigue, and a lack of institutional emphasis on staff rest and recovery.

Conclusions

This audit revealed a high prevalence of staff fatigue and sleep deprivation, with over half of the respondents reporting insufficient sleep prior to shifts. Missed breaks and fatigue-related cognitive lapses were common, and several clinical incidents were retrospectively linked to staff tiredness. Qualitative data highlighted organizational and cultural contributors, including poor rota planning, underreporting, and normalization of exhaustion. The findings support the urgent need for systemic changes in rota design, rest provision, and fatigue risk recognition as part of broader patient safety strategies.

## Introduction

Sleep is a biological necessity for everyone, especially for healthcare workers dealing with life-critical situations. Insufficient sleep and untreated sleep disorders negatively affect healthcare workers’ performance, well-being, and safety [1]. In this context, it also jeopardizes patient safety. The World Health Organization has identified fatigue as a leading factor contributing to medical errors and injuries in healthcare [2].

Sleep deprivation and fatigue among healthcare staff, and their risk to patient safety, represent one of the most sensitive yet vaguely discussed topics in healthcare. This may be due to fear of blame and repercussions, cultural stigma, lack of awareness of the impact of fatigue on patient safety, and normalization of long working hours and fatigue among healthcare professionals. Similarly, systemic issues such as staffing shortages and inadequate scheduling may also play a role.

Anecdotal reports and preliminary observations have raised concerns about frequent missed breaks, insufficient rest before shifts, and increasing staff burnout. However, no formal audit has been conducted to quantify the extent of sleep deprivation and fatigue among staff or to evaluate their impact on patient safety.

Bell et al. [3] stated that insufficient sleep and rest can lead to sleep deprivation and fatigue, predisposing healthcare professionals to errors such as administering incorrect medications, missing early warning signs in patients, making documentation errors, and having slower reaction times in emergencies, all of which jeopardize patient safety.

There are many causes of insufficient sleep and rest among individuals. However, this study specifically explores the work-related causes of sleep deprivation and fatigue in the healthcare sector, particularly among junior staff, their relationship to patient safety risks, and ways to mitigate these factors.

Healthcare staff who work extended hours, consecutive shifts, and mixed patterns of day, twilight, and night shifts within the same week are more likely to experience inadequate rest and sleep. According to the National Institute for Health and Care Excellence (NICE) guidance on shift work and fatigue, shifts should ideally follow a clockwise rotation, moving forward from days to evenings to nights [4].

The National Institutes of Health recommends that adults regularly sleep seven or more hours per night to maintain optimal health. Under the European Working Time Directive, workers must receive a minimum 20-minute break if they work six consecutive hours or more. NICE also emphasizes the importance of protected scheduled naps of less than 30 minutes during work shifts.

Best-practice research from the Health and Safety Executive (HSE) supports the benefits of short sleep, or “power naps,” of around 20 minutes during authorized breaks to help manage fatigue during extended or night shifts [5]. The British Medical Association (BMA) further emphasized the importance of providing proper rest facilities for staff to take such breaks; failure to do so significantly contributes to fatigue-related problems [6].

Several studies have consistently shown that insufficient sleep and fatigue are associated with cognitive impairment, mood alterations, reduced job performance, decreased motivation, and increased safety risks. Alhola and Polo-Kantola [7] reported that both total and partial sleep deprivation cause adverse changes in cognitive performance: total sleep deprivation impairs attention, working memory, and decision-making, while partial sleep deprivation particularly affects vigilance.

Khan and Al-Jahdali [8] further found that sleep deprivation and fatigue are linked to fluctuations in brain activity, including unequal stimulation of the thalamus, default mode network (DMN), amygdala, and hippocampus. These alterations result in impaired attentiveness, memory consolidation, alertness, judgment, and decision-making, among other diminished cognitive functions.

Similarly, Swanson et al. [9] highlighted that poor sleep quality contributes to mental fatigue, which in turn affects executive functions and work performance. Kao et al. [10] also found that sleep quality and fatigue can significantly predict occupational injuries and job-related accidents.

This research is both timely and crucial in addressing the pressing issue of sleep deprivation and fatigue among healthcare staff. It establishes that fatigue is an independent risk factor compromising patient safety and provides guidance on addressing this challenge. By recognizing the risks associated with sleep deprivation and fatigue, healthcare organizations can proactively protect both patients and staff, ultimately improving the overall quality and safety of care.

Aim

The primary goal of this audit is to enhance the well-being of healthcare workers and, in turn, improve patient outcomes. Therefore, this study aims to assess the extent of sleep deprivation and fatigue among healthcare professionals; identify workplace and systemic factors contributing to fatigue; explore its perceived impact on patient safety; provide guidance to mitigate fatigue-related risks in high-acuity clinical settings; raise organizational awareness that fatigue is a modifiable patient safety risk deserving of structured monitoring, policy development, and leadership action; and inform future interventions or quality improvement initiatives.

## Materials and methods

Study design

A cross-sectional audit was conducted over three months across three high-acuity hospital sites within the same NHS trust: Frimley, Wrexham, and Heatherwood. The study employed a non-probability purposive sampling strategy, targeting clinical staff directly involved in inpatient care, including doctors, nurses, and healthcare assistants. Staff were invited to participate via departmental meetings, with the aim of capturing a representative snapshot of fatigue experiences in areas of high clinical intensity and frequent out-of-hours activity.

Participants

Quantitative data were collected from high-acuity wards, including two intensive care units and three high dependency units (surgical and medical acute dependency units), as well as from high-turnover areas such as medical short stay wards and surgical day case wards. Each ward comprised approximately 15-18 junior staff members. Questionnaires were distributed to all available junior staff every Friday over three months. A total of 182 questionnaires were distributed, with 102 returned. Qualitative data were collected through face-to-face interviews offered to junior staff every Wednesday for four weeks, with 87 participants completing interviews.

Data collection

A locally designed, anonymous survey consisting of closed- and open-ended questions was used. Items were developed from NHS workforce well-being tools and fatigue literature (e.g., NHS Staff Survey themes and HSE fatigue indicators) and validated by two clinical governance consultants and one academic lead in workforce health. A pilot study refined question clarity and acceptability. The questionnaire was paper-based, and participation was voluntary, with responses anonymized to promote honesty and minimize bias.

The survey assessed domains including average sleep duration before day and night shifts, frequency of scheduled breaks, self-reported fatigue and its perceived impact on clinical performance, workplace support for rest (including availability of break rooms), experiences of fatigue-related incidents or near misses, and suggestions for reducing fatigue.

Qualitative data were collected through structured face-to-face interviews using a semi-structured guide developed from existing literature and patient safety frameworks. Interviews explored three main questions: how fatigue and sleep deprivation affect patient safety, perceived causes of fatigue, and potential measures to mitigate associated risks. Additional prompts addressed clinical decision-making, communication, workload, rota design, and organizational culture. Interviews lasted approximately five to 10 minutes in private clinical settings. Verbal consent was obtained, and confidentiality was maintained.

Data analysis

Quantitative survey data were analyzed descriptively using frequencies and percentages to explore the prevalence and distribution of fatigue-related experiences. No inferential statistical tests were conducted due to the limited sample size. Qualitative data were manually coded and analyzed thematically to identify recurring patterns, shared experiences, and staff-generated suggestions. This approach provided a nuanced understanding of how fatigue manifests across professional groups and highlighted practical, staff-driven strategies for improving rest, well-being, and patient safety.

## Results

**Figure 1:**
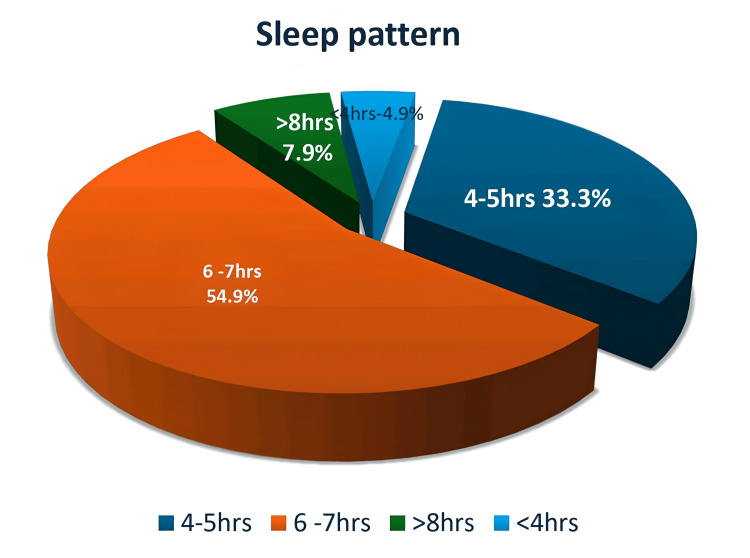
Sleep pattern in percentages

Table 1 presents the data obtained from the anonymous staff survey questionnaires, showing the response categories and the corresponding frequencies and percentages. Figure 1 separately illustrates the distribution of sleep pattern responses in percentages.

**Table 1:** Survey results on fatigue and sleep deprivation among healthcare staff

Category	Response	Frequency	Percentage
Sleep pattern	<4 hours	5	4.9%
	4-5 hours	33	33.3%
	6-7 hours	56	54.9%
	>8 hours	8	7.9%
Working fatigued	0 shifts	13	12%
	>1	89	88%
Well rested before work	Always	3	4%
	Often	11	10%
	Rarely	88	86%
Scheduled breaks	Always	13	12.7%
	Occasionally	34	33.65%
	Rarely	46	45%
	No breaks	9	8%
Proper break stations	Designated rest station	10	10%
	No designated stations	66	65%
	Not applicable	26	25%
Clinical errors related to fatigue	Confirmed	40	39%
	Denied	46	45%
	Preferred not to say	16	16%
Fatigue-related symptoms	Yes	102	100%
Comfortable raising concerns	Comfortable	41	40%
	Uncomfortable	26	25%
	Unsure	35	35%

The findings revealed concerning patterns of sleep deprivation and fatigue among healthcare staff. Only 7.9% of respondents reported getting more than seven hours of sleep per night, while the majority (54.9%) averaged six to seven hours. A further 33.3% reported only four to five hours, and 4.9% slept less than four hours.

Regarding fatigue at work, 86% of respondents admitted to working more than one shift while sleep-deprived or exhausted, whereas only 13% reported never having done so. Similarly, only 4% felt consistently well-rested before work, while 86% stated they were rarely rested.

Break patterns were also suboptimal: just 12.7% always took their scheduled breaks, 32.3% occasionally did, 44.1% rarely did, and 8% reported not having breaks at all. Only 10% of respondents reported having a designated rest station on their ward, while 65% indicated that such facilities were unavailable. When asked about fatigue-related clinical incidents, 39% confirmed they had witnessed or made an error in which fatigue was a contributing factor, 45% had not, and 16% preferred not to disclose.

All respondents (100%) experienced at least one fatigue-related symptom, including difficulty concentrating, delayed responses to patients, forgetfulness, irritability, or poor communication. Regarding workplace culture, 40% felt comfortable raising concerns about fatigue, 25% were uncomfortable, and 35% were unsure. Staff suggestions for reducing fatigue included ensuring uninterrupted and regular breaks, providing proper rest pods, especially for night shifts, improving rota design to avoid mixed day-night weeks, involving staff in scheduling decisions to enhance flexibility and fairness, promoting an open culture around fatigue reporting, fostering teamwork, and addressing staffing levels to reduce workload pressure.

Qualitative data: description, themes, and patterns

In addition to quantitative measures, the audit incorporated semi-structured face-to-face interviews with 87 healthcare professionals across different clinical grades and disciplines. The interviews explored three main questions: how fatigue and sleep deprivation among healthcare staff affect patient safety, what the perceived causes of fatigue and sleep deprivation are, and what measures could mitigate these risks.

Thematic analysis was performed on the qualitative data using an inductive approach. Transcripts and written responses were reviewed independently and coded manually to identify commonalities, patterns, and recurring ideas. Five dominant themes emerged:

Impaired Clinical Performance and Safety Concerns

Several participants reported experiencing fatigue-related cognitive and physical impairments, including slowed thinking, reduced attention, irritability, and decreased motivation. Some openly admitted to near-miss incidents or reduced quality of patient interactions when fatigued, highlighting the direct impact on clinical performance and patient safety.

Normalization of Fatigue

Many staff described tiredness as an expected part of the job, particularly among junior staff and shift-based workers. This cultural acceptance of fatigue contributed to underreporting and reluctance to raise concerns, even when exhaustion negatively affected clinical performance.

Lack of Rest Facilities and Protected Breaks

Respondents highlighted the absence of suitable rest areas, especially during night shifts. Staff frequently missed or shortened breaks due to high patient acuity, understaffing, or fear of judgment from colleagues.

Education on the Risks of Fatigue and Sleep Deprivation

Most respondents emphasized the need to incorporate discussions on fatigue and rest into staff safety briefings, debriefs, and handovers, as well as into mandatory training and induction programs. Leadership messaging that validates fatigue as a patient safety concern, rather than a personal weakness, was also highlighted as essential.

Proper Rest Facilities and Protected Breaks

While compliance with BMA/NHS UK standards for rota design was not formally evaluated, 38% of surveyed staff reported that working three consecutive night shifts left them fatigued and sleep-deprived by the third day, which corresponded with most errors occurring on that day. Similarly, 40% of staff working day shifts reported that consecutive long shifts left them tired and unable to achieve adequate rest on their days off, contributing to fatigue-related performance issues.

## Discussion

This study highlights the prevalence of sleep deprivation and fatigue among healthcare staff and their direct and indirect impact on patient safety. The data reveal that only 7.9% of staff achieve more than seven hours of sleep per night, while the majority (88.2%) obtain between four and seven hours. Such inadequate sleep is known to impair cognitive function and increase the risk of clinical errors. In a web-based survey, Barger et al. [11] reported that interns committed significantly more fatigue-related medical errors resulting in adverse patient outcomes during months with five or more overnight call shifts, compared with months without extended shifts. These errors were linked to inadequate sleep, poor sleep quality, sleep deprivation, and fatigue.

Notably, 86% of staff admitted to working more than one shift while sleep-deprived or fatigued, with only 13% reporting that they consistently felt well-rested before work. Booker et al. [12] noted that this is unsurprising, as healthcare is highly demanding, and the primary issue with shift work and long hours is the resulting tiredness, which impairs the ability to function and focus on work. According to the Health Services Safety Investigations Body, fatigue can have physical, psychological, and emotional effects that negatively influence performance, decision-making, and overall functioning [13].

Break adherence further compounds this issue. Only 12.7% of staff consistently took their scheduled breaks, while almost half (44.1%) reported rarely taking breaks, and 8% reported never taking any. Although skipping breaks to meet the demands of fast-paced clinical environments, such as emergency departments, may be common, long periods without rest can impair cognitive and motor performance to a degree comparable to alcohol intoxication [14].

This chronic rest deficit is often exacerbated by environmental limitations, as only 10% of staff reported having access to a proper rest station in their wards. A study by Fatigue Science confirms that providing dedicated spaces for rest during breaks is a practical way to mitigate fatigue, particularly during long shifts or periods of overtime when errors can have serious consequences [15]. On-site rest facilities equipped with comfortable seating, quiet areas, and sleep pods can offer essential recovery time, reduce errors, improve focus, and boost morale.

All respondents reported experiencing fatigue-related symptoms at work, including difficulty concentrating, delayed patient responses, forgetfulness, and poor communication. These outcomes are not merely well-being issues; they represent tangible threats to patient safety. Alarmingly, 39% of staff confirmed witnessing or being involved in a fatigue-related clinical error, while 16% chose not to disclose.

The culture surrounding fatigue is another concern, as only 40% of staff felt comfortable raising fatigue-related concerns, with the remainder either unsure or uncomfortable. Perceiving fatigue as a personal failing rather than a safety concern exacerbates underreporting. It is crucial to reassure staff that reporting tiredness is professional, promote a supportive environment where staff will not be blamed for disclosing fatigue, and act on reports by ensuring visible follow-up, such as rota adjustments and break facilitation. Sharing examples of change resulting from staff reports and recognizing those who raise concerns constructively can build trust and reinforce the importance of addressing fatigue to protect patients.

Cultural normalization of exhaustion, as echoed in interviews, discourages transparency and suppresses early intervention. Key contributing factors identified included long, unpredictable shifts, missed breaks, rota inflexibility, and fear of being perceived as weak or incapable.

Several practical, staff-driven solutions emerged from the survey, including uninterrupted breaks, proper rest pods, especially during night shifts, improved rota design to avoid mixing day and night shifts in the same week, and involving staff in scheduling decisions. These suggestions align with evidence-based strategies to reduce fatigue, enhance team resilience, and improve patient safety.

The findings demonstrate a significant disconnect between organizational intent and operational reality. Fatigue is currently undermanaged and underrecognized as a clinical risk. To protect patient safety and support staff, healthcare organizations must adopt a systemic approach, such as combining policy, environment, and culture, to address fatigue as a core component of workforce safety.

Recommendations

Education on fatigue management should be embedded as a core component of staff well-being and patient safety initiatives across all clinical areas. Targeted training should be provided for staff working night shifts, long hours, or rotating schedules, focusing on the physiological impact of disrupted circadian rhythms, strategies for safe recovery sleep, and practical methods to manage fatigue during extended shifts. In addition, general education on sleep hygiene should be offered to all shift-working staff, emphasizing the importance of consistent sleep routines, optimal sleep environments, and lifestyle adjustments to improve rest quality.

Fatigue risk awareness should be formally included in staff induction programs to ensure that new employees understand its significance from the outset and reinforced through regular safety huddles and departmental meetings. Fatigue must be acknowledged as a formal patient safety risk and treated with the same seriousness as other hazards, such as medication errors or infection control breaches. To mitigate risk in real time, each ward should display visible break scheduling boards that promote accountability for rest periods and ensure staff receive their entitled, uninterrupted breaks during shifts.

The physical work environment should also support rest and recovery, with designated rest stations available in all clinical areas, equipped with comfortable seating, hydration options, and a quiet atmosphere conducive to brief restorative breaks. Workforce planning should prioritize predictable and balanced rotas, ensuring sufficient rest periods between shifts and avoiding excessive consecutive night duties. Furthermore, a just and open culture must be cultivated, in which staff feel safe and supported to report fatigue-related concerns or near-miss incidents without fear of blame or stigma.

Through comprehensive education, environmental adjustments, and organizational commitment, fatigue management can be integrated into everyday practice, enhancing staff well-being, sustaining clinical performance, and ultimately improving patient safety outcomes.

Strengths and limitations

This study demonstrates several notable strengths. The use of a mixed-methods approach, combining quantitative surveys and qualitative face-to-face interviews, provided a comprehensive understanding of staff experiences, behaviors, and perceptions related to fatigue. By engaging directly with frontline healthcare workers across diverse roles, the study captured rich, first-hand insights into how fatigue affects patient safety and clinical performance. Its practical relevance is particularly strong, as it addresses a significant and modifiable issue within clinical governance and patient safety frameworks. Additionally, the use of confidential and anonymized data collection methods encouraged participants to share honest reflections without fear of judgment or consequence, enhancing the reliability and authenticity of responses on such a sensitive topic.

However, the study also has several limitations. The absence of objective fatigue measurements, such as actigraphy, sleep tracking, or cognitive performance testing, means that findings are based on subjective perceptions rather than direct physiological data. The relatively short audit period may limit the generalizability of results, as it does not capture seasonal variations in workload, rota patterns, or staff availability. Furthermore, potential interviewer bias cannot be excluded, as participants may have modified their responses during face-to-face interviews due to perceived power dynamics or familiarity with the interviewer. Lastly, reliance on self-reported data introduces the risk of recall bias and social desirability bias, with some staff potentially underreporting the extent of fatigue or its impact on clinical performance.

## Conclusions

This audit demonstrates that sleep deprivation and fatigue are highly prevalent among healthcare staff working in high-acuity clinical environments, with more than half reporting inadequate sleep and many struggling to access protected breaks or appropriate rest facilities. These conditions were associated with measurable impacts on clinical performance, including reduced concentration, slowed decision-making, and increased risk of errors or near misses. The qualitative data underscored a deeply rooted cultural normalization of exhaustion, where staff accept fatigue as an unavoidable part of their role and feel unable or unsupported to report concerns. The review of incident reports further highlighted that fatigue is an underrecognized contributor to patient safety events, suggesting that current reporting systems do not adequately capture this risk. Overall, the findings reinforce the urgent need for organizational strategies that promote rest, improve rota design, enhance staff awareness of fatigue risks, and integrate fatigue into patient safety frameworks. Addressing these systemic issues is essential to safeguarding both staff well-being and the quality of care.

## Appendices

Anonymous Staff Questionnaire

Staff Sleep and Fatigue Survey - Improving Patient Safety

This anonymous survey is part of a quality improvement audit looking at how staff fatigue and sleep deprivation may impact patient safety. Your honest input will help guide practical changes. All responses are confidential.

Section 1: About you (optional)

1.    What is your role?

o    Doctor

o    Nurse

o    HCA

2.    What is your typical shift pattern?

o    Days only

o    Nights only

o    Rotating day and night

o    Long shifts (>12 hours)

o    Short shifts (<8 hours)

Section 2: Sleep patterns

1.    On average, how many hours of sleep do you get before a shift (Multiple choice)

o    Less than 4 hours

o    4-5 hours

o    6-7 hours

o    8 hours or more

2.    In the past seven days, how many shifts did you work while feeling sleep-deprived or excessively tired? (Multiple choice)

o    0

o    1-2

o    3-4

o    5 or more

3.    How often do you feel well-rested when coming to work? (Linear scale: 1-5)

1 - Never

2 - Rarely

3 - Sometimes

4 - Often

5 - Always

Section 3: Breaks and rest

1. Do you consistently take your scheduled breaks during shifts? (Multiple choice)

o    Yes, always

o    Most of the time

o    Occasionally

o    Rarely

o    No

2. Do you have access to a quiet or rest space during night shifts? (Multiple choice)

o    Yes

o    No

o    Not applicable

Section 4: Patient safety

1. Have you made or witnessed a clinical error where staff fatigue was a contributing factor? (Multiple choice)

o    Yes

o    No

o    Prefer not to say

2. In the past month, have you experienced any of the following due to tiredness at work?

o    Difficulty concentrating

o    Delayed response to patient needs

o    Forgetting tasks or medications

o    Irritability or poor communication

o    None of the above

3. Do you feel comfortable raising concerns about staff fatigue and patient safety in your team?

o    Yes

o    No

o    Unsure

Section 5: Final thoughts

1.    What do you think would help reduce fatigue among staff on your ward/unit?

2.    Any additional comments on how sleep deprivation affects your work or patient care?

Thank you.
